# Targeting the ubiquitin system by fragment-based drug discovery

**DOI:** 10.3389/fmolb.2022.1019636

**Published:** 2022-09-27

**Authors:** Cassandra Kennedy, Katherine McPhie, Katrin Rittinger

**Affiliations:** Molecular Structure of Cell Signalling Laboratory, The Francis Crick Institute, London, United Kingdom

**Keywords:** ubiquitin, drug discovery, fragment-based drug discovery, chemical tools, ubiquitination

## Abstract

The ubiquitin system contains a wealth of potential drug targets for many diseases and conditions, including neurodegenerative, immune, metabolic and developmental diseases, as well as multiple cancers. Despite years of research, relatively few clinical inhibitors or specific chemical probes for proteins within the ubiquitin system exist, with many interesting target proteins yet to be explored. Fragment-based drug discovery (FBDD) offers efficient and broad coverage of chemical space with small libraries, using covalent and non-covalent approaches. Coupled with advances in structural biology and proteomics, FBDD now provides a thorough screening platform for inhibitor discovery within the ubiquitin system. In this mini review, we summarise the current scope of FBDD and how it has been applied to ubiquitin-activating (E1), ubiquitin-conjugating (E2), ubiquitin ligase (E3) and deubiquitinating (DUB) enzymes. We also discuss the newest frontiers of FBDD and how they could be applied to enable inhibitor and novel chemical probe discovery and provide functional insight into the ubiquitin system.

## Introduction

**FIGURE 1:**
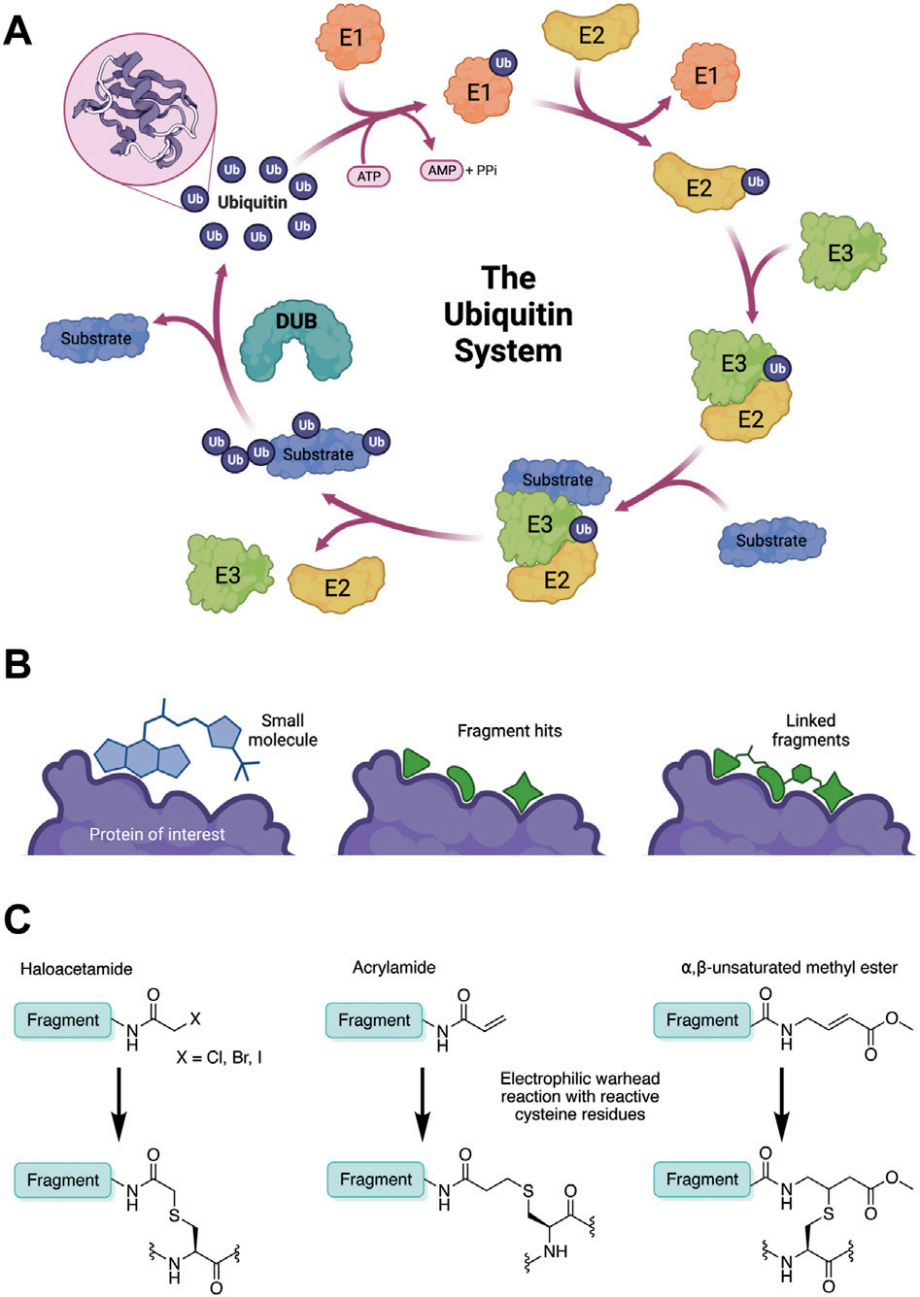
**(A)** The ubiquitin system involves a cascade of E1 (orange), E2 (yellow) and E3 (green) enzymes to add ubiquitin (purple) to the substrate protein (blue). DUB enzymes (teal) can remove ubiquitin from the substrate. **(B)** FBDD can better identify binding hot spots in pockets than traditional small molecule screens. Fragments can then be linked together to create molecules with higher binding affinities. **(C)** Common cysteine-reactive warheads for covalent fragment screens include halo-acetamides, acrylamides and α,β-unsaturated methyl esters.

Ubiquitination is a post-translational modification (PTM) which regulates the majority of cellular processes, from protein degradation and homeostasis to cell cycle control and immune signalling (Komander and Rape, 2012). Proteins are marked with single molecules of ubiquitin (Ub) or poly-ubiquitin chains, *via* surface lysine residues. Ubiquitination is mediated by an ATP-dependant enzymatic cascade of E1-activating, E2-conjugating and E3 ligase enzymes. Proteins containing ubiquitin-binding domains (UBDs) recognise the modification; and proteases called deubiquitinating enzymes (DUBs) cleave ubiquitin chains. Together, this cellular machinery comprises the ubiquitin system (Figure 1A).

The diversity and complexity of the ubiquitin code is vast. Poly-ubiquitin chains have multiple conformations: chains can be homotypic, mixed or branched, and each promote different cellular outcomes. For example, Lys48-linked chains mark proteins for proteasomal degradation, whereas Lys63-linked chains activate immune signalling pathways (Swatek and Komander, 2016). Furthermore, recent studies have shown that ubiquitination is not limited to lysine residues and can even occur on other biomolecules (Otten et al., 2021; Squair and Virdee, 2022). Specificity of chain type and substrate recognition comes from the specific E2/E3 combination used. Dysregulation of the ubiquitin system is a driver for many different diseases, and so targeting the ubiquitination machinery offers attractive therapeutic opportunities, as well as scope for tool compound development to better understand cellular function and disease development (Wertz and Wang, 2019).

With over 600 E3 ligases, around 100 DUBs (Harrigan et al., 2018), 40 E2 enzymes, and only two E1 enzymes known in humans (Jin et al., 2007), each class of enzyme offers both advantages and disadvantages as targets for tool compound development and therapeutic intervention. Several small molecule inhibitors of the proteasome, E1s and DUBs are progressing through clinical trials (Wang et al., 2016; Hyer et al., 2018), however few exist as approved drugs (Manasanch and Orlowski, 2017; Wertz and Wang, 2019). Achieving specific responses to inhibition of the proteasome and E1 enzymes is difficult since both have such broad reactivities and form so many interactions. For inhibiting specific cellular pathways, the E3 and DUB families are the most favourable drug targets within the ubiquitin system, however few E3 ligases or DUBs have modulators in the clinic or are liganded (Wu et al., 2020).

### Fragment-based drug discovery

Traditional high throughput screening (HTS) methods for drug discovery involve screening libraries of hundreds of thousands of small molecules against a protein of interest or a phenotype (MacArron et al., 2011). While this approach has successfully identified many lead compounds for many different targets, there are key limitations. The relatively large and complex “drug-like” molecules used in HTS often form sub-optimal interactions with the target protein due to steric hinderance and conformational inflexibility. This means that other, stronger interactions may be masked, resulting in an inferior starting point for hit-to-lead optimisation (Figure 1B). Furthermore, due to the structural complexity of these “drug-like” molecules, very large libraries are required to attain sufficient hit rates. Fragment-based drug discovery (FBDD) has emerged as an alternative hit-finding method in the last two decades (Erlanson et al., 2016). In FBDD, much smaller molecules (“fragments”) are screened, with molecular recognition between optimal pharmacophores and small protein pockets more likely to be identified. The decreased molecular size enables greater coverage of chemical space with a much smaller library of compounds (Kirsch et al., 2019), therefore screening is faster and cost effective. Although this decreased molecular size limits the strength and specificity of target-fragment interactions, this can be regained through fragment elaboration and targeted medicinal chemistry campaigns after hit identification (Figure 1B). Furthermore, fragment hits typically have a much higher ligand efficiency than traditional HTS “drug-like” hits, so desirable physicochemical properties can be maintained during hit-lead optimisation (Kirsch et al., 2019). More recently, covalent fragment screening has developed as an additional modality, where the weaker target-fragment interactions are stabilised by covalent bond formation (Keeley et al., 2020).

Fragments physiochemical properties are typically guided by the “rule of 3”: molecular weight <300 Da, logP ≤3, and fewer than 3 hydrogen-bond donors, hydrogen-bond acceptors and rotatable bonds (Congreve et al., 2003; Jhoti et al., 2013). Non-covalent and covalent fragments require different considerations when developing fragment campaigns against a certain target or phenotype, particularly in library design and detection method. Traditional HTS detection methods, such as *in vitro* activity assays (enzymatic inhibition, or ELISA), are less applicable to fragment campaigns since higher compound concentrations are required to compensate for the lower affinities of fragments compared to small molecules, and this can lead to assay interference (Inglese et al., 2007).

### Non-covalent fragments

The key advantage of non-covalent fragments is that target proteins are not required to have a readily accessible reactive residue. Unless some inherent structural library bias is desired, for example if the target protein favours a particular chemical motif, the library should be designed such that the broadest coverage of chemical space is achieved. Some libraries, such as the Diamond-SGC-iNEXT Poised (DSi-Poised) library, contain fragments which can be easily conjugated post-screening (Cox et al., 2016), or can be grown in multiple vectors (Bancet et al., 2020; Consortium et al., 2021). In addition, libraries can be simplified and streamlined by removing enantiomeric compounds.

Techniques for non-covalent fragment hit identification can be protein-based or ligand-based. In the case of protein-based detection, identification of hits relies on the biophysical properties of the protein target to change upon fragment binding; alternatively, NMR can be used for ligand-based detection. The most common biophysical detection techniques include differential scanning fluorimetry (DSF), nuclear magnetic resonance (NMR) and surface plasmon resonance (SPR) (Kirsch et al., 2019; Gao et al., 2020). More recently, combining hit identification directly with structural insight by X-ray crystallography has become popular, as demonstrated by the development of the XChem platform at Diamond Light Source (DLS) (Douangamath et al., 2021). Fragments are particularly amenable for crystallographic screening: smaller library size ensures practical aspects such as crystal mounting (which is yet to be automated) are manageable, and high fragment solubility ensures the required concentrations can be soaked into the crystal.

Once fragment hits have been identified, typically, a follow-up screen is performed to investigate structure-activity relationships (SAR). Lead fragments are then grown, merged or linked into larger molecules which retain the optimal binding efficiency of the original fragments, but with increased specificity and affinity for the desired protein target. Access to structures of fragment-protein complexes is often necessary to aid the synthetic elaboration of fragments by informing on suitable vectors to optimise target-fragment interactions. Crystallographic fragment screening platforms such as XChem provide this structural insight in a high throughput manner, and have greatly expedited the FBDD process.

### Covalent fragments

In contrast to non-covalent fragments, covalent fragments contain a reactive functional group (often referred to as a “warhead”) in addition to the molecular pharmacophore. As such, covalent fragment engagement is first driven by molecular recognition between pharmacophore and target protein, followed by covalent bond formation between the electrophilic “warhead” and a nearby nucleophilic residue. This additional functionality increases the complexity of the library design, as variations in the electrophile also need to be considered (Lu et al., 2021). Cysteines are the most commonly targeted nucleophilic residues within the proteome; they are the most reactive at physiological pH, and are often key to enzyme catalytic activity, for example in cysteine proteases and some DUBs and E3 ligases. For cysteine-targeting, commonly used electrophiles include α,β-unsaturated methyl esters (Kathman et al., 2015; Johansson et al., 2019), chloroacetamides and acrylamides (Resnick et al., 2019) (Figure 1C). A balance between reactivity and specificity is required, and acrylamides and chloroacetamides are often chosen for this reason. More recently, novel electrophiles have been developed for targeting residues beyond cysteine, including lysines, tyrosines and histidines (Hacker et al., 2017; Ward et al., 2017; Abbasov et al., 2021; Zanon et al., 2021). The main advantage of covalent fragment screening over non-covalent screening is the increased simplicity of hit detection. Hit fragments form a covalent bond with their target residue, resulting in a change in molecular weight which can be easily detected by liquid chromatography coupled mass spectrometry (LC-MS) for purified proteins, and with LC-MS/MS for cell lysates.

FBDD has become increasingly reliable as a drug discovery method, with multiple examples of FDA approved compounds derived from initial fragment hits (Erlanson et al., 2016), including BRAF inhibitor, Vemurafenib (Bollag et al., 2012), and BCL-2 inhibitor, Venetoclax (Souers et al., 2013). In addition, there are several general efforts to identify probes for the entire human proteome (Arrowsmith et al., 2015; Drewes and Knapp, 2018; Carter et al., 2019), towards which FBDD will undoubtedly contribute. Developing high-quality tool compounds to better understand molecular systems and provide insight into protein pathways is becoming commonplace before starting expensive drug campaigns, and FBDD could help to expand the number of probes available. Overall, FBDD is proving to be an incredibly powerful technique for drug and probe discovery, and its application to the ubiquitin system is only just beginning. A summary of the fragment campaigns discussed in the following sections is given in Table 1.

**TABLE 1:** Summary of fragment hits and fragment-derived modulators of ubiquitin system proteins. Pharmacophores from original fragment hits which were retained in optimised compounds are highlighted in blue/green/purple. Potency/binding affinity of optimised compounds was defined individually in each report (see Citation).

Fragment hit	Target	Enzyme class	Fragment binding mode	Detection method and hit rate	Optimised compound	Citation
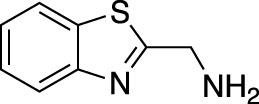	Ube2T	E2	Non-covalent	DSF (3.5%)	NR^a^	Morreale et al. (2017a)
				BLI (7.5%)		
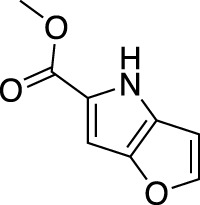 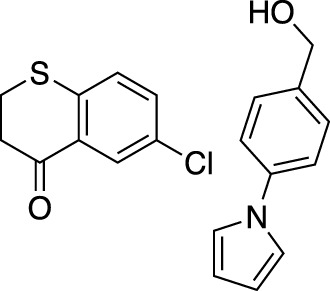	VHL	CRL2 (RING) E3	Non-covalent	DSF (5.2%)	NR^a^	Lucas et al. (2018)
	
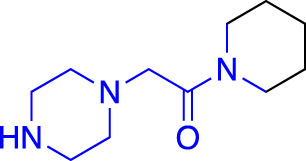	XIAP/cIAP	RING E3	Non-covalent	NMR	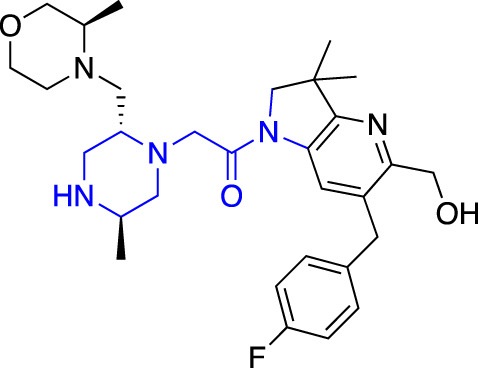	Chessari et al., (2015); Tamanini et al., (2017)
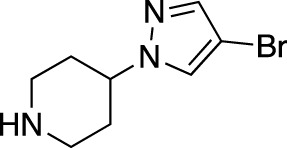						
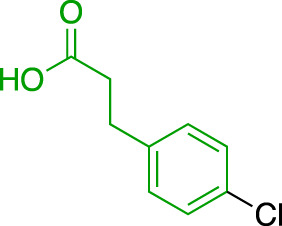 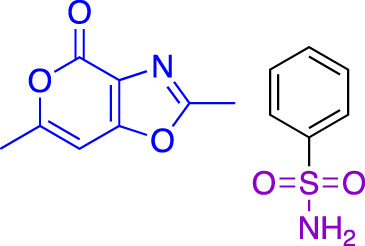	KEAP1	CRL3 (RING) E3	Non-covalent	X-ray Crystallo-graphy (NR^a^)	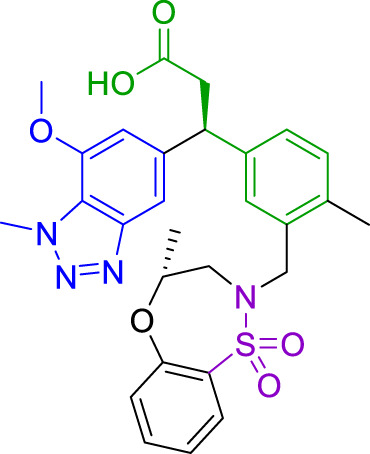	Davies et al., (2016); Heightman et al., (2019)
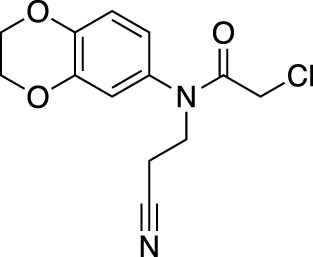	FEM1B	CRL2 (RING) E3	Covalent (chloro-acetamide)	FP (NR^a^)	NR^a^	Henning et al. (2022b)
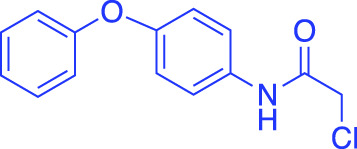	RNF4	RING E3	Covalent (chloro-acetamide)	ABPP (3.5%)	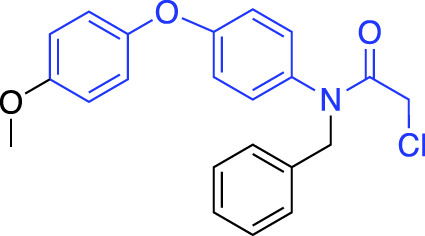	Ward et al. (2019)
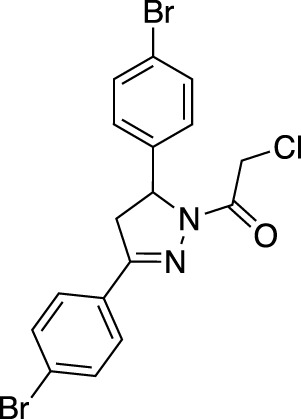	RNF114	RING E3	Covalent (chloro-acetamide)	ABPP (NR^a^)	NR^a^	Luo et al. (2021)
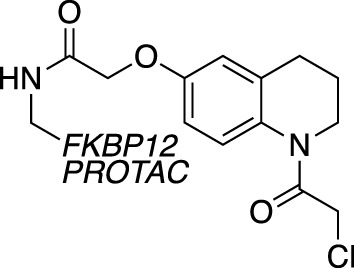	DCAF16	CRL4 (RING) E3	Covalent (chloro-acetamide)	ABPP (N/A^b^)	NR^a^	Zhang et al. (2019)
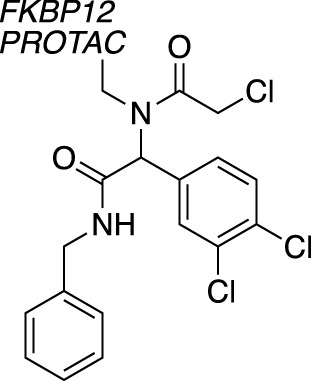	DCAF11	CRL4 (RING) E3	Covalent (chloro-acetamide)	ABPP (N/A^b^)	NR^a^	Zhang et al. (2021)
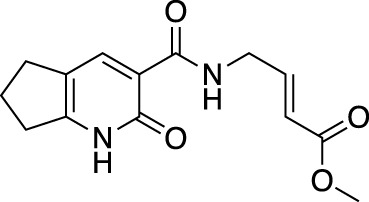	HOIP	RBR E3	Covalent (α,β-unsaturated methyl ester)	LC-MS (NR^a^)	NR^a^	Johansson et al. (2019)
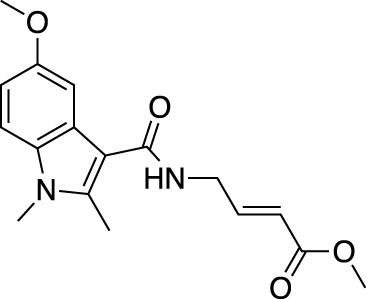	Nedd4-1	HECT E3	Covalent (α,β-unsaturated methyl ester)	LC-MS (2.0%)	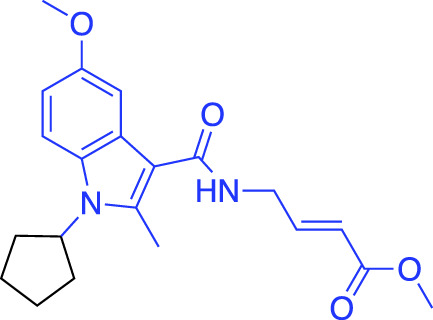	Kathman et al. (2015)
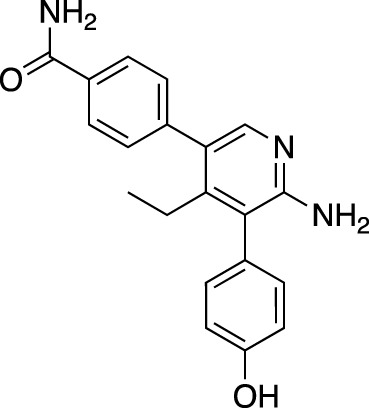	USP7	DUB	Non-covalent	NMR (NR^a^)	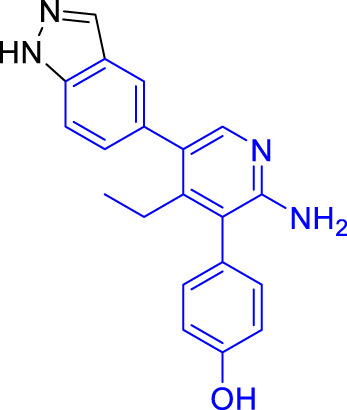	di Lello et al., (2017); Kategaya et al., (2017)
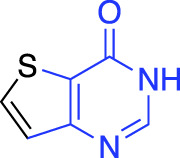 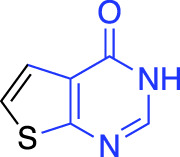	USP7	DUB	Non-covalent	SPR (0.1%)	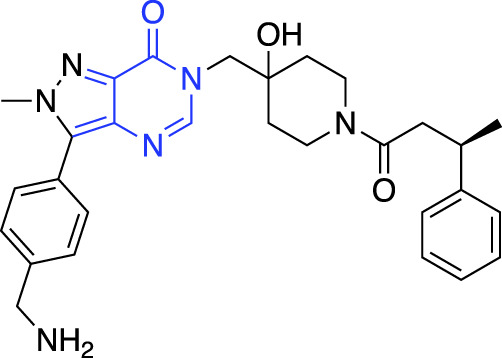	Gavory et al. (2018)
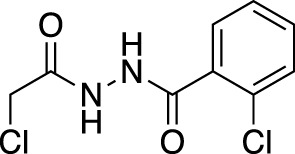 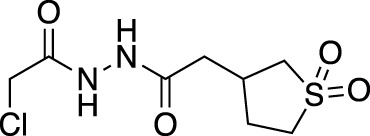	OTUB2	DUB	Covalent (chloro-acetamide)	LC-MS/MS^c^ (4.7%)	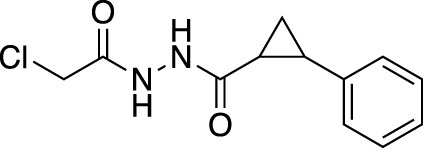	Resnick et al. (2019)
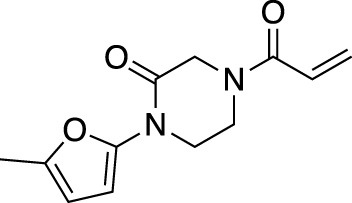	OTUB1	DUB	Covalent (acrylamide)	ABPP (0.1%)	NR^a^	Henning et al. (2022a)

a Not reported.

b Not applicable as scout fragments coupled to FKBP12 binding ligand were used (PROTACs), not direct fragment screening.

c LC-MS/MS proteomics-based fragment screening.

### Ubiquitin-activating (E1) and ubiquitin-conjugating (E2) enzyme fragment-based drug discovery

While E1-activating enzymes are the least specific component of the ubiquitin system, a handful of E1 enzyme inhibitors have been published. E1 inhibitors can be targeted to the ATP binding site, and therefore show exquisite selectivity over other ubiquitin system proteins which are ATP-independent and typically do not have existing small molecule binding sites. One example, small molecule UBE1 inhibitor, TAK-243, showed promise as an anti-tumour agent *in vivo* (Hyer et al., 2018), suggesting that despite risks of high toxicity, E1 proteins could be interesting targets. No fragment campaigns have yet been reported against E1 enzymes, even though any new tool compounds would enable researchers to better understand the role of E1 enzymes in the ubiquitin system and related diseases.

Due to their limited specificity, not many E2 inhibitors currently exist. However, whilst using FBDD to screen the E2 enzyme Ube2T, a novel allosteric binding site was identified (Morreale et al., 2017a). Following a 1200-fragment screening campaign using both DSF and biolayer interferometry (BLI) to identify hits, NMR spectroscopy and X-ray crystallography were used for further validation. This allosteric site provided opportunities for novel inhibitor development and insights into allosteric modulation mechanisms for Ube2T. However, following conflicting SAR results, further biophysical and structural analysis ultimately identified that binding from a zinc impurity was responsible for the inhibition observed (Morreale et al., 2017b).

### Ubiquitin ligase (E3) fragment-based drug discovery

E3 ligases determine substrate specificity as well as chain linkage specificity (either directly, or through E2 selection) and are therefore highly valuable drug targets. As the largest family of enzymes within the ubiquitin system, new tool compounds and probes targeting E3 ligases are needed to better understand their biology and physiological roles. Binders for E3 ligases can be distinguished between inhibitors, which inactivate the enzymes, and recruiters, which are commonly used for targeted protein degradation (TPD), including modalities such as proteolysis-targeting chimeras (PROTACs) (Sakamoto et al., 2001).

The E3 ligase family was originally subdivided into three main classes: Homologous with E6-associated protein C-terminus (HECT), Really Interesting New Gene (RING) and Ring-between-Ring (RBR) (Komander and Rape, 2012; Morreale and Walden, 2016) but more recently, further classes of E3 ligases including RING-Cys-Relay (RCR) enzymes have been identified (Pao et al., 2018; Otten et al., 2021). Of the 600 E3s known, the majority belong to the RING class; only 28 are HECT E3 ligases and 14 belong to the RBR class (Chaugule and Walden, 2016; Morreale and Walden, 2016; de Cesare et al., 2018). These classes have two distinct mechanisms of action. HECT and RBR E3 ligases facilitate transfer of ubiquitin to the target by first accepting a molecule of ubiquitin from the E2-Ub conjugate, *via* a transthiolation reaction forming an E3-Ub thioester bond, before final transfer of ubiquitin to the target. This E3-Ub conjugation occurs on the catalytic cysteine residue in the HECT, RBR, or RCR E3 active site (Walden and Rittinger, 2018). Conversely, RING E3 ligases do not possess this active site cysteine. Instead, they mediate ubiquitin transfer by acting as a scaffold, binding both the E2-Ub conjugate and the target substrate, bringing them into close proximity. The E3 stabilises the active E2-Ub conformation, thus priming the complex for final ubiquitin transfer onto the target substrate (Berndsen and Wolberger, 2014).

Non-covalent and covalent fragment campaigns against RING, HECT, RBR and newly-discovered E3 ligases could yield starting motifs for both inhibition and TPD recruitment purposes, depending on the location of the binding site. However, for the development of inhibitors, the active site cysteine of HECT, RBR and RCR E3 ligases offers an excellent target, thus cysteine-reactive fragment campaigns may be advantageous. Recent work to understand the “ligandable cysteinome” through chemoproteomics suggested that 51 E3 ligases would be susceptible to specific probe design. Interestingly, not all the “ligandable” residues identified were catalytic cysteines, suggesting that covalent fragment screening approaches should not be disregarded for RING E3 ligases (Belcher et al., 2021).

### RING ubiquitin ligases

Within the RING E3 ligases, the largest subclass are the Cullin-RING ubiquitin ligases (CRL) (Nguyen et al., 2017). Differentiation and substrate specificity within this subclass relies on adaptor proteins and substrate receptors, for example Von Hippel Lindau (VHL), cereblon (CRBN) and Kelch Like ECH Associated Protein 1 (KEAP1). It is these appended adaptors which have been the targets of most RING E3 ligase ligand efforts (Rothweiler et al., 2022).

The most studied of the liganded E3 ligases is VHL, a CRL2 E3 ligase, which has a robust and well-used small molecule ligand targeting the VHL:HIFα protein-protein interaction (PPI) interface (Galdeano et al., 2014). Fragment campaigns using FBDD and *in silico* screening approaches have identified several novel VHL binding pockets (Lucas et al., 2018). After a 1200-fragment screen by DSF and NMR, 82 hits were validated by NMR and crystallography, which revealed 18 fragments as true hits. Displacement assays using an HIFα peptide were carried out, highlighting allosteric binding for 17 of the fragments, which was further confirmed by crystallography, and may now form a basis for further tool development.

The CRL3 E3 ligase, KEAP1, was subjected to a screen of 330 non-covalent fragments by crystallography. Key protein-fragment interactions were identified at the KEAP1 interface with its substrate, NRF2 (Davies et al., 2016). Significant fragment elaboration and medicinal chemistry efforts (Heightman et al., 2019) led to a potent compound, KI-696, which showed cellular activity and *in vivo* efficacy, and is now a high-quality chemical probe for the KEAP1-NRF2 PPI interface.

The inducer of apoptosis protein family (IAP) has proved one of the most successfully targeted RING E3 ligase families by FBDD. Previous inhibitors of this family had been peptidomimetic (Sharma et al., 2006) but fragment campaigns identified novel small molecule dual inhibitors of cIAP2 and XIAP (Chessari et al., 2015). 1151 fragments were screened by 1D NMR against XIAP-Bir3, and hits were characterised further using NMR and crystallography. Two overlapping hits demonstrated promising inhibition in fluorescence polarisation (FP) assays and, following significant medicinal chemistry efforts, achieved nanomolar potency and efficacy in mouse models. The elaborated molecule, ASTX660, is now in Phase II trials for the treatment of advanced solid tumours and lymphomas (Chessari et al., 2015; Tamanini et al., 2017).

More recently, covalent FBDD has driven novel ligand discovery for RING E3 ligases. Uncharacteristically, FEM1B, a CRL2 E3 ligase was screened against a covalent cysteine-reactive fragment library using an FP-assay, as opposed to LC-MS. The lead fragment utilised a chloroacetamide electrophile which reacted with a key cysteine residue responsible for substrate recognition (Henning et al., 2022b). The fragment was further developed into a FEM1B recruiter for novel PROTACs, and further SAR and medicinal chemistry is ongoing to develop the lead fragment into a stable and effective tool compound.

RNF4 and RNF114 have also been screened against covalent fragment libraries. Using gel-based activity-based protein profiling (ABPP) with a fluorescent iodoacetamide probe, an RNF4 hit fragment was identified. This fragment bound to two key zinc-coordinating cysteine residues without inhibiting RNF4 auto-ubiquitination activity, making it an ideal ligand for development into a TPD recruiter motif. Following further SAR optimisation, the fragment was developed into a PROTAC for BRD4 degradation but was found to be nonspecific for RNF4 (Ward et al., 2019). Similarly, RNF114 was subjected to a covalent fragment screen using gel-based and proteomics ABPP, and the lead fragment developed into PROTACs for BRD4 and BCL-ABL degradation (Luo et al., 2021).

A novel method, using broadly reactive electrophilic scout fragments (Backus et al., 2016; Bar-Peled et al., 2017) as E3 ligase recruiters in PROTAC molecules, identified two novel CRL4 E3 ligases, DCAF16 and DCAF11, for TPD purposes. Following the identification of a PROTAC which degraded FKBP12, affinity enrichment, proteomics and genomic sequencing were used to validate DCAF16 as the E3 enzyme responsible for the degradation (Zhang et al., 2019). This method identified degraders of specific targets, and then retrospectively deconvoluted the responsible E3 ligase. This contrasts with previously described methods, where fragment screens were used to identify recruiter ligands for specific E3 ligases.

### HECT and RBR ubiquitin ligases

HECT, RBR, and RCR E3 ligases rely on a catalytic cysteine for their activity and are therefore obvious targets for covalent fragment screening to identify novel inhibitors. As these E3 classes are less numerous than RING E3 ligases, there are fewer examples of fragment campaigns against them.

The first example of a covalent fragment screening campaign against an E3 ligase with a catalytic cysteine was the HECT E3 ligase, Nedd4-1. The HECT domain of Nedd4-1 was screened against an α,β-unsaturated methyl ester fragment library by LC-MS (Kathman et al., 2015). Hit fragments were found to bind to a non-catalytic cysteine at the ubiquitin-binding interface and validated by X-ray crystallography. However, at endogenous levels, ubiquitin outcompeted fragment binding. Following SAR optimisation, an N-cyclopentyl substituted fragment derivative was found to be the most potent, whilst retaining specificity. Interestingly, this tool compound was then used to show that Nedd4-1 switches from elongating ubiquitin chains through a processive mechanism to a distributive mechanism when bound to the compound. Previously, it was thought that all HECT E3 ligases were processive, and so this finding provided new insights into the mechanism of HECT E3 ligases, and highlights how FBDD can be used to develop excellent tool compounds for the ubiquitin system.

HOIP RBR E3 ligase is one of the three subunits which comprise the linear ubiquitin chain assembly complex (LUBAC). A fragment library of pooled α,β-unsaturated methyl esters was screened against HOIP using LC-MS to identify hit fragments, which were confirmed to bind to the active site cysteine. Despite structure-based fragment optimisation, efficacy could not be improved significantly, however, inhibition of HOIP and impressive selectivity over other ubiquitin system proteins was demonstrated in cells, suggesting that further medicinal chemistry efforts could yield a potent and specific tool compound (Johansson et al., 2019).

Whilst other HECT and RBR E3 ligases have been liganded through small molecule screening (Watt et al., 2018; Tian et al., 2019), many E3 ligases do not have high-quality chemical probes yet and would be worthy targets for future FBDD campaigns.

### Deubiquitinating (DUB) enzyme fragment-based drug discovery 

DUBs have been implicated in many different disease pathways, including cancers, neurodegenerative diseases, and immunity and infection (Harrigan et al., 2018). A number of DUB inhibitors are in the drug development pipeline (Rowinsky et al., 2020; Schauer et al., 2020), some of which have been discovered through FBDD.

In 2017, USP7 was the first DUB targeted with a non-covalent fragment screen, using NMR as a hit detection method (Kategaya et al., 2017). Lead compounds were assessed using ABPP, and prioritised based on their specificity, biophysical properties and toxicity in multiple cell lines. These hits were subjected to *in silico* development and medicinal chemistry, resulting in small molecule inhibitors of USP7 (di Lello et al., 2017). USP7 was also subjected to a non-covalent fragment screen using SPR (Gavory et al., 2018). Hit fragments were compared to known inhibitors of USP7, and a stereocentre discovered to be key to activity of the fragments. Crystallography was then used to understand and optimise fragment-protein interactions, before biochemical and biophysical analysis showed excellent selectivity of the final compound for USP7 against other related DUBs. The final compound showed efficacy in cells, resulting in stabilisation of p53 and decreased levels of MDM2.

More recently, electrophilic covalent fragment screening against recombinant proteins with mass spectrometry was combined with high-throughput crystallography (Resnick et al., 2019). A panel of clinically relevant proteins, including DUBs OTUB2 and USP8 were screened, and hits were identified by LC-MS. 47 fragment hits were identified for OTUB2, which were further developed into a lead series, guided by crystallography. Hits were validated for selectivity using cell and lysate gel-based and proteomics ABPP experiments in HEK293T cells, highlighting the power of combining mass spectrometry with high throughput crystallography for FBDD.

Chemical biology has seen a huge increase in PROTAC development, with the recruitment of a range of novel E3 ligases demonstrated, such as for RNF4 (Ward et al., 2019) and FEM1B (Henning et al., 2022b). Excitingly, the first deubiquitinase-targeting chimera (DUBTAC) was recently reported (Henning et al., 2022a), where DUBs are recruited to new targets for targeted protein stabilisation. A cysteine-reactive fragment campaign identified specific recruiters for DUB proteins through gel-based ABPP. A fragment hit for OTUB1 was further developed and coupled with a small molecule binder for a mutant CFTR chloride channel to create the first DUBTAC, highlighting how FBDD can be used to make novel tools.

### Future Outlooks

FBDD has enabled the development of novel tool compounds for a range of E1, E2, E3, and DUB proteins. However, many ubiquitin system proteins remain unliganded, and novel technologies are emerging to expand the scope of FBDD.

So far, covalent fragment-based campaigns have been focused on targeting cysteine residues. However, other nucleophilic residues beyond cysteine are more prevalent, and more likely to be post-translationally modified *in situ*. Profiling these residues with fragments could provide interesting insights into biology and highlight new drug targets. Despite their lower intrinsic reactivity, advances have been made in targeting lysine residues with sulfotetrafluorophenyl ester electrophiles (Hacker et al., 2017; Abbasov et al., 2021), and serine, threonine, tyrosine and histidine residues with N-hydroxysuccinimide esters (Ward et al., 2017; Zanon et al., 2021). In addition, profiling of cysteines, lysines, tyrosines and histidines has been characterised with sulfonyl fluorides (Gilbert et al., 2022), and tyrosines with sulfur-triazole exchange chemistry (Brulet et al., 2020). These exciting advances in non-cysteine residue profiling have yet to be fully translated into FBDD but are likely to be at the forefront of the field in the future.

Photoaffinity labelling (PAL), a technique initially developed for target identification (Smith and Collins, 2015), has also been utilised for FBDD. A library of highly reactive carbene intermediates was formed *in situ* using UV irradiation of diazirine precursors. The carbene inserts into proximal bonds within 15 Å, forming a covalent bond, even when there is no nucleophilic residue available. This has so far been reported with recombinant proteins (Grant et al., 2020) and directly in cells (Bar-Peled et al., 2017). Despite challenges with non-specific crosslinking and low crosslinking yields, the technology remains a powerful method to identify novel tool compounds for the ubiquitin system.

Recently, metallofragments have been highlighted as a promising new avenue for FBDD. Using a metal ion to coordinate fragments adds 3D character and increases shape diversity in library design. This could be particularly useful for target systems where metallo-compounds have already shown efficacy, such as antibacterial compounds (Hess, 2022). A novel metallofragment library has already shown activity and specificity against a few protein targets (Morrison et al., 2020), but has yet to be screened against ubiquitin system proteins.

We believe that the newest, cutting-edge discoveries in FBDD will be seen through the development and use of fragment campaigns in collaboration with phenotypic screening. Advances in proteomics methods for reactive residue profiling have been reported recently, for example by the Gygi and Cravatt groups (Roberts et al., 2017; Maurais and Weerapana, 2019; Kuljanin et al., 2021). By coupling reactive residue profiling with covalent fragment screening in relevant cellular models for diseases of interest, researchers will be able to identify fragment and residue pairs on a proteome-wide level and identify whether they affect the disease phenotype. If this method is applied to the ubiquitin system, we could rapidly see a rise in specific and potent chemical tools and therapeutic molecules entering drug development, enabling researchers to better understand this complex and dynamic signalling pathway.
